# Hydatid Cyst Disease of the Thyroid Gland: A Rare Case Report

**DOI:** 10.5812/ijem-163775

**Published:** 2025-07-31

**Authors:** Pooneh Dehghan, Fatemeh Ghiasi, Seyyed Hasan Langari, Hossein Moradkhani

**Affiliations:** 1 Department of Radiology, School of Medicine, Ayatollah Taleghani Hospital, Shahid Beheshti University of Medical Sciences, Tehran, Iran

**Keywords:** Thyroid Hydatid Cyst, *Echinococcus granulosus*, Zoonosis, Thyroid Gland, Thyroidectomy

## Abstract

**Introduction:**

Hydatid cysts caused by *Echinococcus granulosus* are zoonotic infections endemic to pastoral regions. While hepatic (50 - 70%) and pulmonary (20 - 30%) involvement dominate, primary thyroid hydatidosis is exceptionally rare (< 1% of cases), posing diagnostic and therapeutic challenges. This case underscores the importance of considering parasitic etiologies in thyroid nodules, particularly in endemic zones.

**Case Presentation:**

A 26-year-old female patient presented with complaints of pain and swelling in the anteroposterior region of the neck, predominantly on the right side. Fine needle aspiration (FNA) from the nodule seen on ultrasound (US) of the thyroid gland was reported to be suspicious for papillary thyroid carcinoma (PTC), and the patient underwent bilateral total thyroidectomy. In the postoperative histopathological examination, a hydatid cyst was confirmed in the thyroid gland.

**Conclusions:**

Thyroid hydatid cysts, though rare, require high clinical suspicion in endemic regions. Imaging (US/MRI) and serology are pivotal for preoperative diagnosis, while FNA is contraindicated. Complete surgical excision with adjuvant albendazole ensures optimal outcomes. Public health measures, including dog deworming and community education, are critical to disrupting the parasite’s lifecycle.

## 1. Introduction

Hydatid disease, caused by *Echinococcus granulosus*, is a zoonotic infection with a worldwide distribution, particularly endemic in pastoral regions such as the Middle East and parts of Asia, Africa, and South America (1, 2). The liver (50 - 70%) and lungs (20 - 30%) are the most frequently involved organs, while thyroid involvement is extremely rare, accounting for less than 1% of all reported cases (3, 4).

The clinical importance of thyroid hydatid cysts lies in their ability to mimic common thyroid pathologies, including colloid nodules, cystic papillary thyroid carcinoma (PTC), or necrotic lymph nodes, often leading to misdiagnosis and unnecessary aggressive surgical procedures. Because fine needle aspiration (FNA) is contraindicated due to the risk of anaphylaxis and dissemination, diagnosis before surgery remains challenging (5).

Herein, we present the case of a 26-year-old female from an endemic region who developed progressive neck swelling and pain. Despite initial suspicion of PTC on ultrasound (US) and FNA, postoperative histopathology revealed a hydatid cyst of the thyroid gland. The uniqueness of this case is twofold: Its extreme rarity and the fact that a misdiagnosis resulted in an unnecessary total thyroidectomy instead of a more conservative procedure.

The aim of this case report is to highlight the diagnostic pitfalls, therapeutic challenges, and lessons learned from managing such an uncommon presentation, and to compare our experience with similar cases reported in the literature (6-8).

## 2. Case Presentation

**Figure 1.**
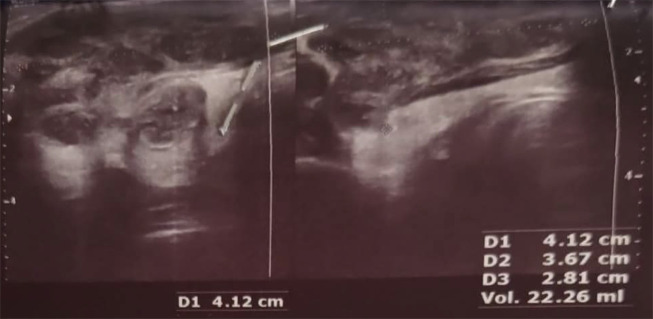
A hypoechoic lesion containing fluid with internal echoes and thick internal septations, measuring 41 × 36 × 28 mm in size and 22 cc in volume, is seen in the upper outer aspect of the right lobe.

**Figure 2.**
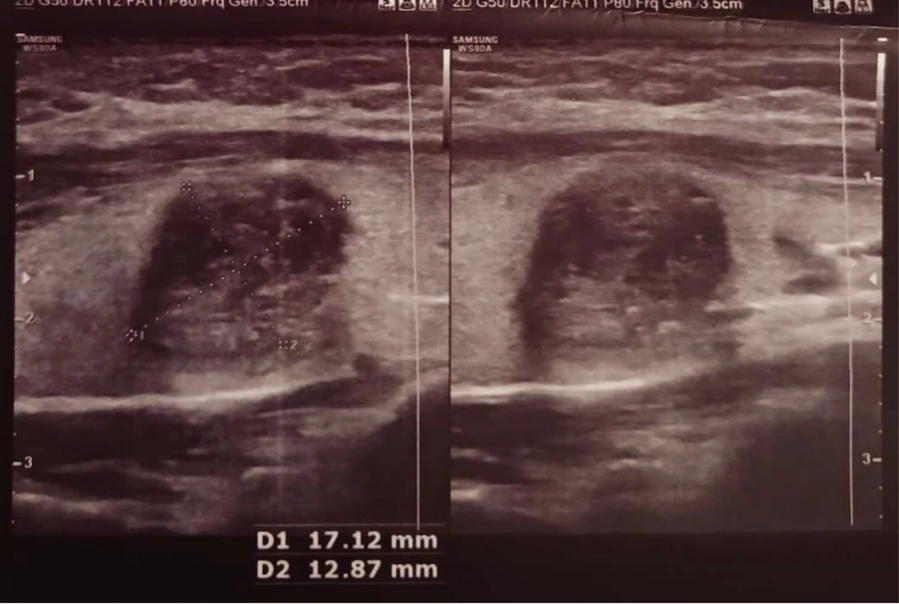
A hypoechoic, wider-than-tall nodule with smooth borders and peripheral and punctate calcifications measuring 17 × 13 mm is seen in the right lobe of the thyroid gland (TIRADS = 5). According to the fine needle aspiration (FNA) performed on the nodule, it was reported as suspicious for papillary thyroid carcinoma (PTC).

**Figure 3.**
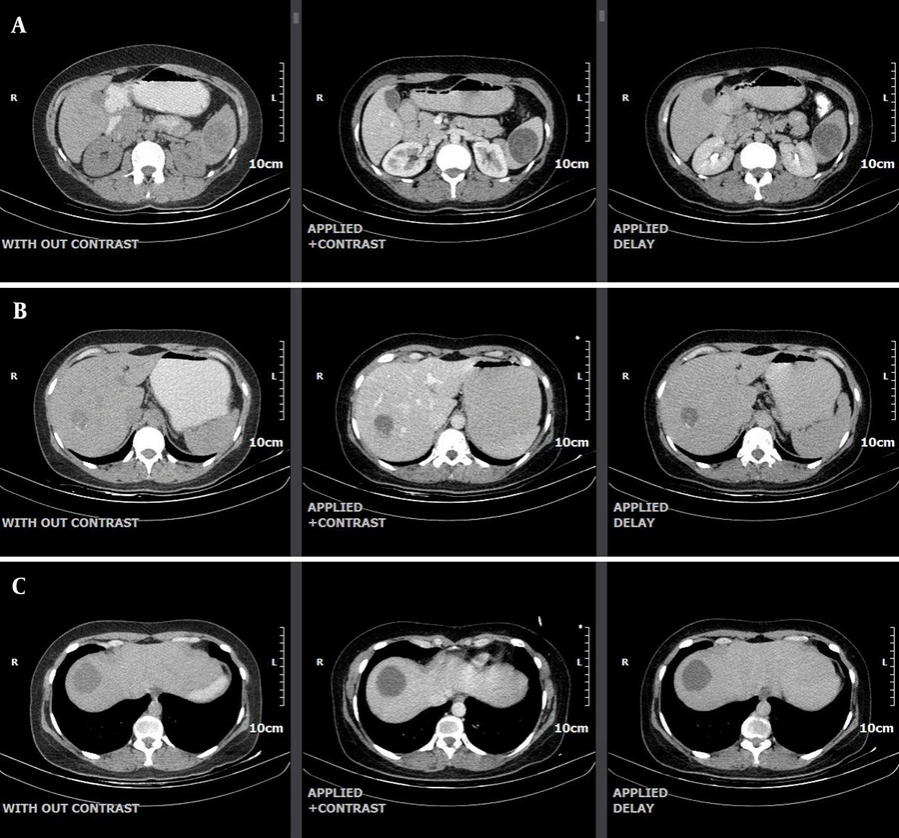
A, computed tomography (CT) images demonstrate a hydatid cyst measuring 43 mm in diameter in the spleen; B, computed tomography (CT) images demonstrate a hydatid cyst measuring 32 mm in diameter in segment 7 of the liver; C, computed tomography (CT) images demonstrate a hydatid cyst measuring 38 mm in diameter in segment 8 of the liver.

A 26-year-old female patient presented to our hospital with a 3-month history of progressive pain and swelling in the anterosuperior neck, predominantly on the right side. On the patient’s physical examination, a soft, mobile nodule approximately 3 × 2 cm in size was palpated in the right lobe of the thyroid gland. Cervical lymphadenopathy was detected, and examinations of other systems were normal. Blood tests and thyroid tests were within normal limits. The thyroid US revealed a hypoechoic, fluid-filled lesion with internal echoes and thick internal septations, measuring 41 × 36 × 28 mm and with a volume of 22 cc, suggestive of a conglomerate necrotic lymph node (Level III-IVa, Figure 1). Additionally, a hypoechoic nodule, wider than tall, with well-defined borders and peripheral and punctate calcifications measuring 17 × 13 mm was observed (TIRADS V, Figure 2). The FNA of this nodule was suspicious for PTC. Consequently, the patient underwent bilateral total thyroidectomy. No intraoperative and postoperative complications developed. After surgery, the patient was prescribed albendazole 40 mg twice a day, and her symptoms improved. The patient is being followed up without any recurrence. A CT scan of the chest, abdomen, and pelvis performed after total thyroidectomy revealed evidence of hydatid cysts in the liver and spleen but no hydatid cysts in the lungs (Figure 3A - C ).

## 3. Discussion

Primary hydatid disease of the thyroid gland is extremely rare, with fewer than 100 cases documented worldwide (2, 3). This rarity, combined with nonspecific clinical and imaging features, creates significant diagnostic challenges. In our patient, the lesion was initially suspected to be PTC, leading to a total thyroidectomy rather than a conservative approach. The final diagnosis of a hydatid cyst was established only by postoperative histopathology.

### 3.1. Diagnostic Strengths and Limitations

**Figure 4.**
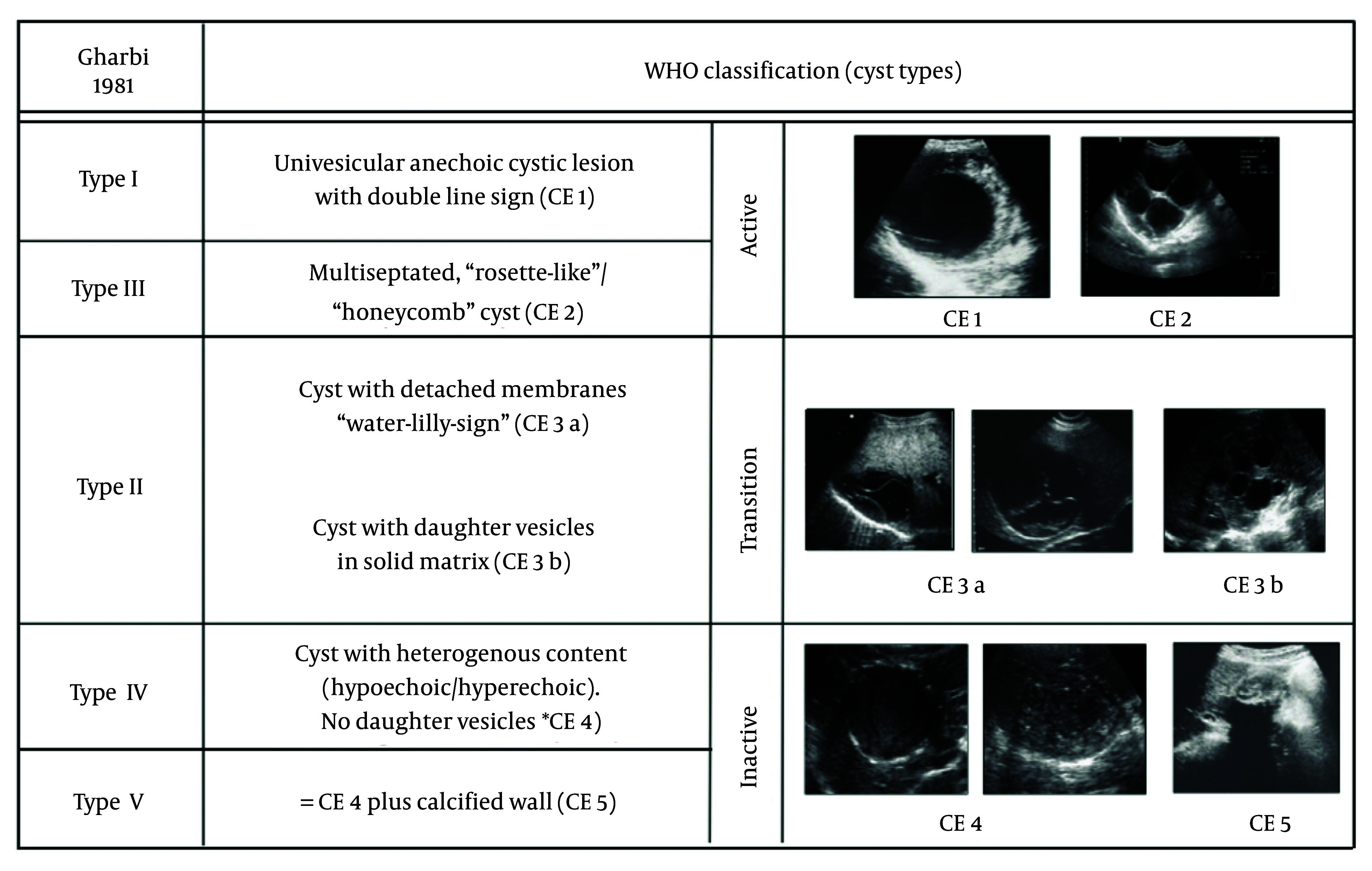
WHO ultrasound (US) classification of hydatid cysts

The diagnostic workup of thyroid hydatid cysts is particularly challenging due to the absence of pathognomonic findings. The US is the first-line imaging tool in thyroid evaluation and may occasionally reveal features such as the “cyst-within-cyst” or “water lily sign”, which are considered suggestive of hydatidosis (9). However, these findings occur in fewer than 40% of cases. In our patient, the lesion mimicked a necrotic lymph node and a suspicious nodule (TIRADS V), leading to an erroneous suspicion of malignancy (Figure 4). 

The FNA, although highly valuable in routine thyroid nodule evaluation, is contraindicated in suspected hydatid disease because of the risk of spillage, dissemination, and anaphylaxis (10). In this case, FNA not only posed a potential risk but also misled the diagnostic process by suggesting PTC. Cytopathology, although useful in differentiating neoplastic from benign lesions, is incapable of reliably identifying hydatid structures, particularly in isolated thyroid disease. Serological assays, including ELISA and immunoblot, can provide supportive evidence and have reported sensitivity ranging from 60% to 90% (11). Nevertheless, their role in isolated thyroid hydatidosis is limited, and false negatives are common in calcified or inactive cysts. In our patient, serology was not performed preoperatively; however, positive results combined with the detection of hepatic and splenic cysts might have raised earlier suspicion of hydatid disease and altered the surgical plan.

### 3.2. Misdiagnosis and Unnecessary Surgery

The suspicion of malignancy prompted a total thyroidectomy in this case. While thyroidectomy is justified for confirmed cancers, in hydatid disease it represents overtreatment, exposing the patient to unnecessary surgical risks and obligating lifelong hormone replacement. If the correct preoperative diagnosis had been made, the patient could have undergone simple cyst enucleation or partial thyroidectomy, which preserve thyroid function and carry a lower risk of complications (12). This case illustrates how overlooking hydatid disease in the differential diagnosis of thyroid nodules may result in unnecessary radical surgery.

### 3.3. Role of Extra-Thyroid Cysts in Diagnosis

Another critical aspect of this case was the later identification of hydatid cysts in the liver and spleen, which were only discovered after thyroidectomy. Hydatid disease most commonly involves the liver (50 - 70%) and lungs (20 - 30%), while splenic involvement is less frequent (6, 7). Detection of these lesions preoperatively might have supported suspicion of systemic echinococcosis and prompted reconsideration of the thyroid lesion as a hydatid cyst. In patients from endemic areas, whole-body imaging should be considered when thyroid cystic lesions present with atypical features and malignancy cannot be confirmed with certainty.

### 3.4. Comparison with Literature

Several cases of thyroid hydatid cysts have been reported in the literature, most of which highlight the same diagnostic difficulties. Safarpour et al. (2) described two cases of thyroid hydatid cysts misdiagnosed as malignant nodules, with the correct diagnosis established only postoperatively. Akbulut et al. (3) similarly reported preoperative misdiagnosis and emphasized that clinical and radiological findings are insufficient for accurate identification. The World Health Organization has stated that rare cases and suggested that the thyroid microenvironment may hinder cyst maturation, explaining the extreme rarity of this condition. Nonetheless, when thyroid involvement occurs, it frequently mimics carcinoma, as in our case. Kesici (12) reported a case series where enucleation was successfully performed with preservation of thyroid function. Compared to their report, our case demonstrates the adverse consequences of misdiagnosis, namely unnecessary radical surgery and lifelong hormone replacement. Taken together, these studies support the conclusion that awareness of hydatid disease as a differential diagnosis for thyroid nodules is crucial in endemic regions.

### 3.5. Conclusions

Hydatid disease of the thyroid gland is an exceptionally rare condition that can mimic malignant thyroid nodules, leading to misdiagnosis and unnecessary radical surgery. This case highlights several important lessons: (1) Clinicians in endemic areas should maintain a high Index of Suspicion for hydatid cysts when evaluating atypical or cystic thyroid nodules, particularly when imaging findings are inconclusive; (2) FNA, although routine for thyroid nodules, may be misleading and poses a risk in suspected hydatid disease; (3) comprehensive systemic imaging and serological evaluation are critical to identify multi-organ involvement and to guide an accurate diagnosis; and (4) enucleation or limited thyroid surgery is preferable to total thyroidectomy when hydatid disease is suspected.

This case underscores the importance of integrating epidemiological context, imaging findings, and cautious diagnostic interpretation. More broadly, the lessons learned can be applied to the evaluation of cold thyroid nodules in endemic regions, reminding clinicians that parasitic diseases, though rare, should remain part of the differential diagnosis.

### 3.6. Strengths 

The strength of this report lies in its detailed description of a misdiagnosed thyroid hydatid cyst and the clinical consequences of unnecessary radical surgery. By documenting this case and comparing it with similar reports, we provide valuable lessons for clinicians in endemic areas.

### 3.7. Limitations

The limitations include the absence of preoperative serological testing and systemic imaging, which could have contributed to an earlier suspicion of hydatidosis. Additionally, as with any single case report, the findings cannot be generalized universally; however, they reinforce the importance of epidemiological context, comprehensive imaging, and cautious interpretation of thyroid nodules in endemic regions. Timely diagnosis and education are very effective ways to treat many diseases (13, 14).

## Data Availability

The dataset analyzed during the present study is available from the corresponding author on reasonable request. However, the data are not publicly available due to ethical restrictions and patient confidentiality, in accordance with institutional and regulatory guidelines.
